# The potential of PIVKA-II as a treatment response biomarker in hepatocellular carcinoma: a prospective United Kingdom cohort study

**DOI:** 10.18632/oncotarget.28136

**Published:** 2021-11-23

**Authors:** Vandana M. Sagar, Kathyrn Herring, Stuart Curbishley, James Hodson, Peter Fletcher, Salil Karkhanis, Homoyon Mehrzad, Pankaj Punia, Tahir Shah, Shishir Shetty, Yuk Ting Ma

**Affiliations:** ^1^Institute of Immunology and Immunotherapy, University of Birmingham, Birmingham, UK; ^2^The Cancer Centre, University Hospitals Birmingham NHS Foundation Trust, Birmingham, UK; ^3^Institute of Translational Medicine, University Hospitals Birmingham NHS Foundation Trust, Birmingham, UK; ^4^Cancer Research UK Clinical Trials Unit, University of Birmingham, Birmingham, UK; ^5^Department of Radiology, University Hospitals Birmingham NHS Foundation Trust, Birmingham, UK; ^6^The Liver Unit, University Hospitals Birmingham NHS Foundation Trust, Birmingham, UK; ^*^These authors contributed equally to this work (joint first authors); ^#^These authors contributed equally to this work (joint senior authors)

**Keywords:** hepatocellular carcinoma, biomarker, PIVKA-II

## Abstract

Prothrombin induced by vitamin K absence II (PIVKA-II) has recently been validated internationally as a diagnostic biomarker for hepatocellular carcinoma (HCC), as part of the GALAD model. However, its role as a treatment response biomarker has been less well explored. We, therefore, undertook a prospective study at a tertiary centre in the UK to evaluate the role of PIVKA-II as a treatment response biomarker in patients with early, intermediate and advanced stage HCC. In a cohort of 141 patients, we found that PIVKA-II levels tracked concordantly with treatment response in the majority of patients, across a range of different treatment modalities. We also found that rises in PIVKA-II levels almost always predated radiological progression. Among AFP non-secretors, PIVKA-II was found to be informative in 60% of cases. In a small cohort of patients undergoing liver transplantation, pre-transplant PIVKA-II levels predicted for microvascular invasion and poorer differentiation. Our results demonstrate the potential utility of PIVKA-II as a treatment response biomarker and in predicting microvascular invasion, in a Western population. PIVKA-II demonstrated improved performance over AFP but, as a single biomarker, its performance was still limited. Further larger prospective studies are recommended to evaluate PIVKA-II as a treatment response biomarker, within the GALAD model.

## INTRODUCTION

Hepatocellular carcinoma (HCC) is the seventh most common cancer and the third most common cause of cancer-related death worldwide [1]. Although the burden is highest in East Asia and Africa, the incidence is rising rapidly in the West [2]. The prognosis of HCC is poor, with incidence and mortality rates that are almost equivalent and detecting this tumour at an early stage continues to be a major challenge with the majority of patients presenting with intermediate or advanced stage disease [2, 3].

Current Clinical Practice guidelines recommend HCC surveillance in high risk individuals, to increase the detection of early-stage HCC, increase curative treatments and thus improve survival [4, 5]. However, one of the major limitations of HCC surveillance is the lack of reliable serum biomarkers. Alpha-fetoprotein (AFP) is the most widely used biomarker for HCC worldwide but, due to its suboptimal sensitivity and specificity in surveillance (fluctuating low levels that may be due to flare of viral hepatitis and small proportion of early stage tumours secreting AFP), the previous American Association for the Study of Liver Diseases (AASLD) and the current European Association for the Study of the Liver (EASL) guidelines for surveillance recommend six-monthly ultrasound imaging (US) alone [5, 6]. Despite this, the performance of ultrasound imaging alone is limited, being user-dependent and with reported sensitivity ranging from 65–80% and specificity of over 90% when used as a screening test [6]. There therefore remains an unmet need for better serum biomarkers.

Prothrombin induced by vitamin K absence II (PIVKA-II), also known as des-gamma-carboxyprothrombin (DCP), is another serum biomarker of HCC that was first reported in 1984 [7]. PIVKA-II is an abnormal form of prothrombin which is released into the blood when the vitamin K-dependent post-translational carboxylation of glutamic acid residues is inhibited, for example, due to the absence of vitamin K or in the presence of vitamin K antagonists. It has been extensively studied as a diagnostic biomarker (predominantly in Asia) and subsequent meta-analyses have confirmed it to have a similar sensitivity to AFP for detecting small HCC (<3 cm) but a higher specificity and area under the ROC curve (AUC) compared to AFP [8, 9]. However, the combination of both biomarkers does not appear to significantly improve the diagnostic accuracy of PIVKA-II alone [8, 9].

The BALAD (bilirubin, albumin, Lens culinaris agglutinin-reactive AFP (AFP-L3), AFP and DCP/PIVKA-II) and subsequent GALAD (gender, age, AFP-L3, AFP and DCP/PIVKA-II) scoring systems were developed in an attempt to overcome the insufficient performance of ultrasound imaging for HCC detection, by combining three well characterised and commercially available serum biomarkers (AFP-L3, AFP and DCP/PIVKA-II) [10–12]. The superior performance of the GALAD model for the detection and diagnosis of HCC has recently been confirmed in an international cohort of almost 7000 patients, with 80–91% sensitivity and 81–90% specificity for the detection of early stage HCC [12]. Consequently, measurement of these three biomarkers has now been incorporated into the Japanese Clinical Practice guidelines for HCC surveillance [13].

The landscape of treatment for HCC has changed significantly over the last few years with the approval of several tyrosine kinase inhibitors and immunotherapy, as well as selective internal radiotherapy [5]. Consequently, there is not only an urgent need for better screening tools, but also for biomarkers which will support optimal management and decisions to switch therapy.

Besides diagnosis, the emerging role of PIVKA-II as a biomarker of treatment response and in predicting microvascular invasion has also been reported [14–21]. At the commencement of this study, all of these studies had been performed only in Asia. However, given that PIVKA-II has been shown to perform better in viral aetiologies of cirrhosis, compared to non-viral aetiology, and the 95% reference range for PIVKA-II has been shown to differ significantly between geographical regions in healthy individuals [22–25], it is therefore important to assess this in a Western population too. We therefore undertook a prospective study, at a tertiary UK centre, to evaluate the role of PIVKA-II as a treatment response biomarker and in predicting microvascular invasion.

## RESULTS

A total of 141 patients with hepatocellular carcinoma were enrolled between March 2016 and March 2018. Of these, 90 patients had early stage HCC (Supplementary Figure 1), 16 patients had intermediate stage HCC and 35 patients had advanced stage HCC. Baseline characteristics of these cohorts are summarised in Table 1.

**Table 1:** Baseline characteristics grouped by BCLC stage

	Early stage HCC (BCLC A)	Intermediate stage HCC (BCLC B)	Advanced stage HCC (BCLC C)
	(*n* = 90)	(*n* = 16)	(*n* = 35)
***Demographics***			
Age (Yrs)			
Median	63	62	68
IQR	56–69	52–64	55–73
Sex (%)			
Male	66 (73%)	12 (75%)	28 (80%)
Female	24 (27%)	4 (25%)	7 (20%)
Aetiology (%)			
HBV/HCV	22 (24%)	5 (31%)	8 (23%)
ALD	35 (39%)	5 (31%)	5 (14%)
NAFLD	18 (20%)	1 (6%)	6 (17%)
Other	12 (13%)	2 (13%)	4 (11%)
Unknown	3 (3%)	3 (19%)	12 (34%)
Treatment (%)			
Ablation	26 (29%)	0	0
Transplantation	18 (20%)	0	0
Resection	2 (2%)	0	0
TACE	39 (43%)	16 (100%)	0
Sorafenib	0	0	35 (100%)
None	5 (6%)	0	0
Child Pugh class (%)			
A	85 (94%)	16 (100%)	28 (80%)
B	5 (6%)	0	7 (20%)
C	0	0	0
Portal vein invasion (%)			
Yes	0	0	10 (29%)
***Tumour markers***			
AFP (ng/mL)			
Median	10.2	43.6	56.3
IQR	5.2–84.1	17.0–257.2	8.8–2115.3
Non-secretors (%)	54 (60%)	5 (31%)	8 (23%)
PIVKA-II (mAU/mL)			
Median	170.0	925.8	6430.2
IQR	48.9–591.4	179.2–3287.8	320.0–29306.4
Non-secretors (%)	15 (17%)	1 (6%)	0

Abbreviations: CLD: chronic liver disease; HCC: hepatocellular carcinoma; TACE: transarterial chemoembolisation; IQR: interquartile range; HBV: hepatitis B virus; HCV: hepatitis C virus; ALD: alcoholic liver disease; NAFLD: non-alcoholic fatty liver disease; BCLC: Barcelona Clinic Liver Cancer Classification; AFP: alpha-fetoprotein.

### Baseline PIVKA-II and AFP levels

**Figure 1:**
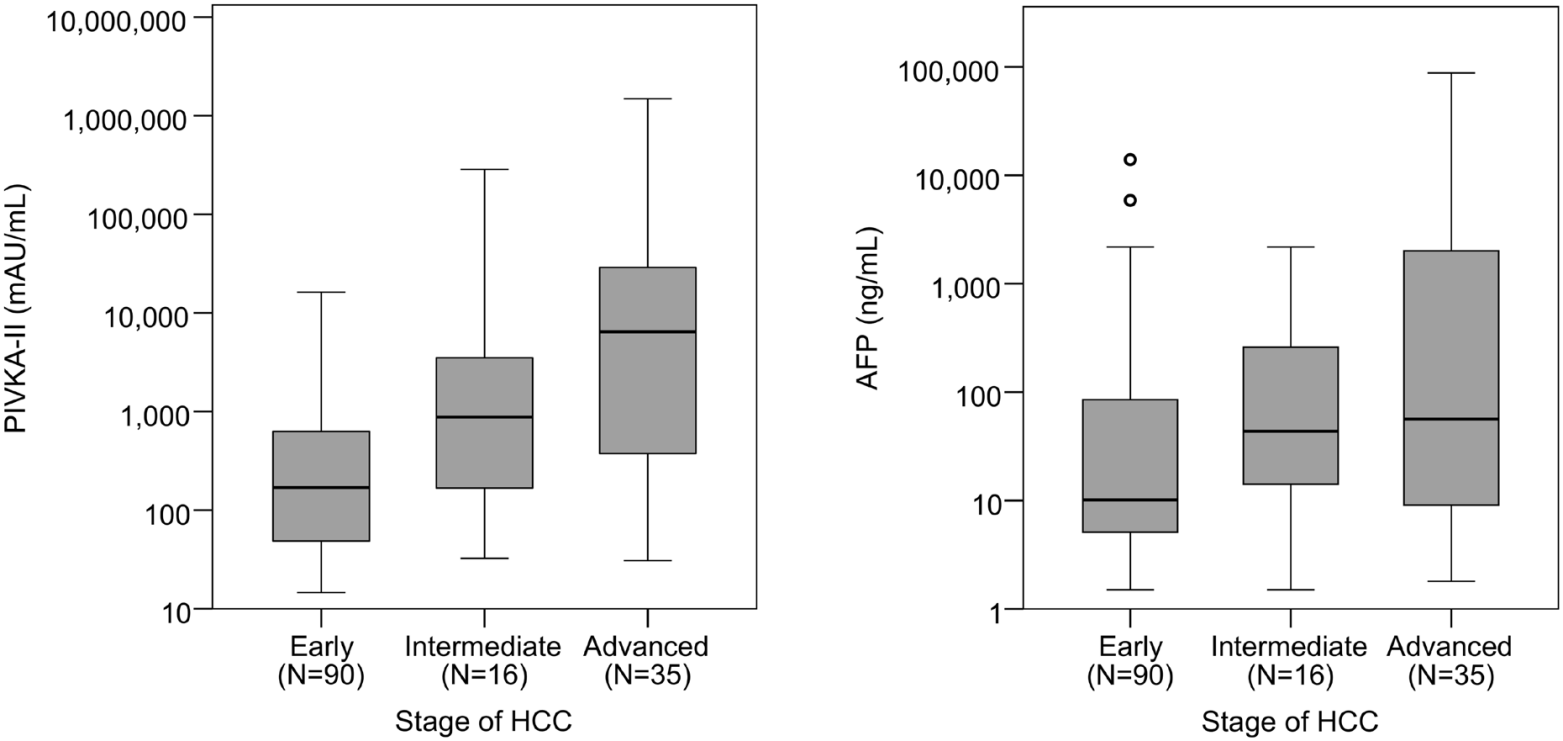
Boxplots of PIVKA-II and AFP by stage of HCC. A log_10_ scale has been used for the PIVKA-II and AFP values on the y-axis. Jonckheere-Terpstra tests found both markers to increase significantly with the stage of HCC (PIVKA-II: *p* < 0.001, AFP: *p* = 0.002).

Baseline (pre-treatment) serum PIVKA-II and AFP levels were measured in all patients. Baseline PIVKA-II and AFP levels increased significantly with tumour stage (Figure 1).

### Correlation with size and number of lesions

**Figure 2:**
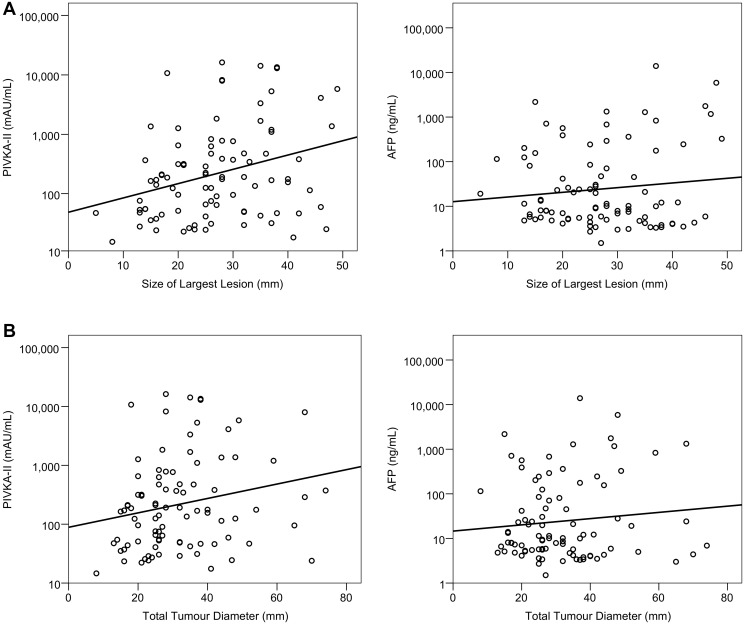
Associations between size of lesions and PIVKA-II and AFP in early HCC. A log_10_ scale has been used for the PIVKA-II and AFP values. Trend lines are from linear regression models, with the log_10_-transformed values of the markers as dependent variables. The largest lesion size was found to be significantly correlated with PIVKA-II (Spearman’s rho:0.295, *p* = 0.005), but not AFP (rho:0.045, *p* = 0.676) (**A**). The total tumour diameter was also significantly correlated with PIVKA-II (Spearman’s rho: 0.256, *p* = 0.015), but not AFP (rho: 0.017, *p* = 0.874) (**B**).

Within the early HCC cohort, the relationships between tumour-related factors and both PIVKA-II and AFP were assessed. PIVKA-II levels showed a significant positive correlation with size of the largest HCC lesion (rho: 0.295, *p* = 0.005; Figure 2A) and total tumour diameter (rho: 0.256, *p* = 0.015; Figure 2B) but not with the number of lesions (rho: −0.018, *p* = 0.865). AFP levels were not significantly associated with the size of the largest HCC lesion (rho: −0.045, *p* = 0.676; Figure 2A), total tumour diameter (rho: 0.017, *p* = 0.874; Figure 2B) or the number of HCC lesions (rho: 0.076, *p* = 0.479).

### Correlation with explant histology

A total of 16 patients underwent liver transplantation for early HCC. In two patients, no cancer was detected on explant histology, so these were excluded from further analysis. Associations between tumour-related factors and both PIVKA-II and AFP were assessed (Table 2).

**Table 2:** Associations between explant histology findings and pre-transplant PIVKA-II and AFP

	*N*	PIVKA-II (mAU/mL)	*P*-value	AFP (ng/mL)	*P*-value
Microvascular Invasion			0.036		0.635
No	10	50.6 (28.7–205.6)		16.0 (5.8–202.8)	
Yes	4	380.5 (206.5–7343.5)		14.2 (3.8–26.0)	
Number of Lesions			0.364		0.240
1	9	205.6 (46.4–472.7)		8.0 (4.2–19.8)	
>1	5	54.8 (28.7–124.6)		24.1 (23.9–27.9)	
HCC Differentiation			0.026		0.198
Well	2	50.6 (46.4–54.8)		9.0 (5.8–12.1)	
Moderately	8	62.8 (28.0–165.1)		13.9 (4.3–25.9)	
Poorly	4	2199.1 (256.5–9162.1)		133.6 (14.2–1001.4)	

Data are reported as median (IQR), with *p*-values from Mann-Whitney *U* tests for comparisons across two groups, or Jonckheere-Terpstra tests for three groups.

Higher pre-transplant PIVKA-II levels were found to be significantly associated with the presence of microvascular invasion on explant histology (*p* = 0.036), whilst no significant association with AFP levels were observed (*p* = 0.635). Higher pre-transplant PIVKA-II levels were also significantly associated with poorer HCC differentiation (*p* = 0.026), whilst no significant association was observed with AFP (*p* = 0.198). There were no significant associations observed between pre-transplant levels of either PIVKA-II or AFP and the number of HCC lesions in the explant.

Five patients also had serum PIVKA-II and AFP levels measured post-transplantation, with a median of 372 days between pre- and post-transplant measurements. Reduction in PIVKA-II levels following transplantation was observed in all five patients, from a median of 124.6 mAU/mL to 38.2 mAU/mL (Wilcoxon’s test: *p* = 0.063). Conversely, serum AFP levels were non-informative, as all five patients were AFP non-secretors.

### PIVKA-II and AFP levels in patients undergoing ablation therapy

A total of 26 patients planned for radiofrequency ablation were enrolled, with follow up samples collected in 13 patients. The baseline samples were taken a median of 35 days prior to ablation and serial PIVKA-II and AFP levels were measured approximately every three months thereafter, to coincide with routine clinic visits, until either recurrence or the end of follow up. The median number of samples collected per patient was 3 (range 2–7).

**Figure 3:**
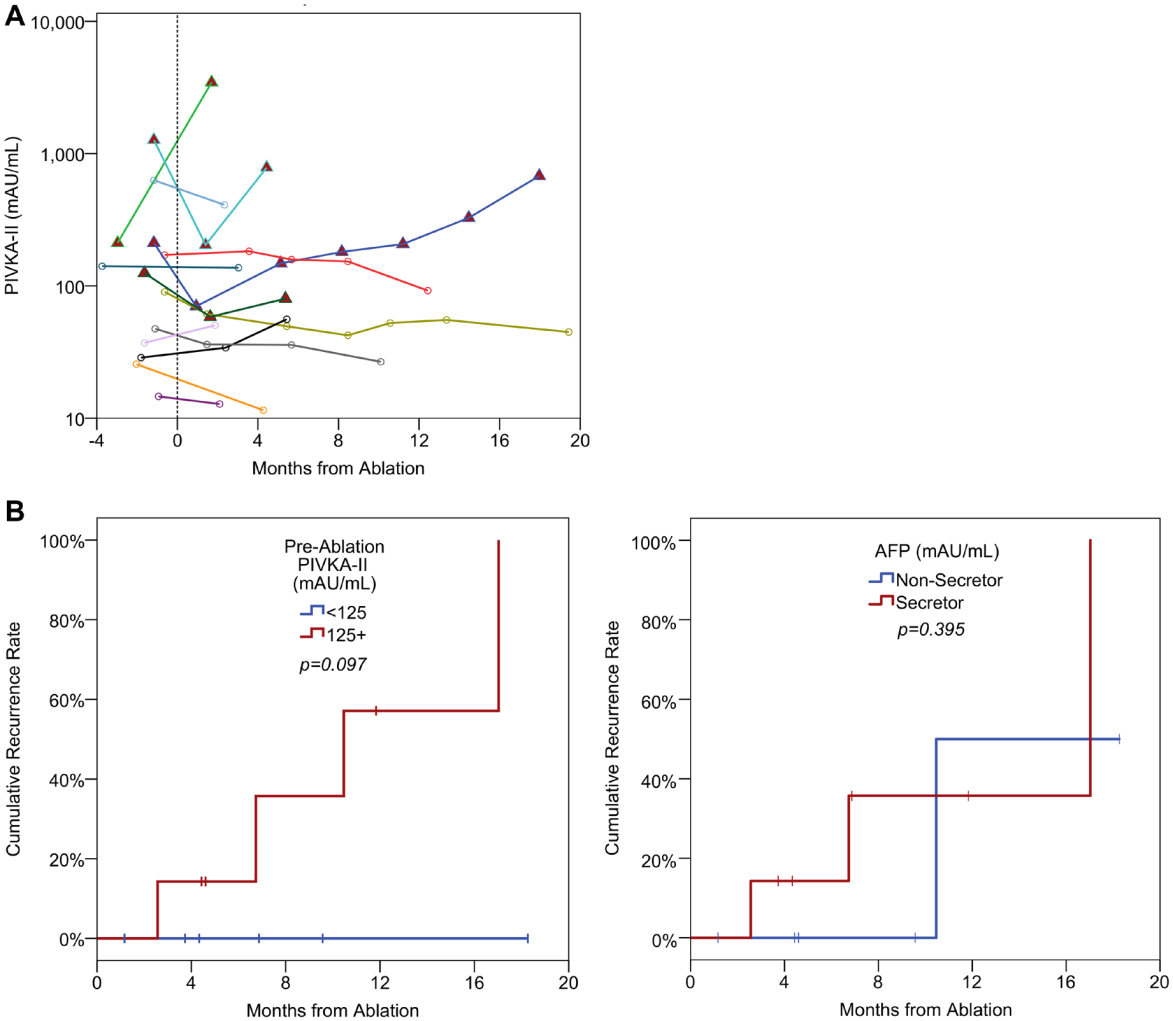
(**A**) Spider plots of longitudinal PIVKA-II levels in ablation patients. A log_10_ scale has been used for the PIVKA-II values. The broken vertical line represents the timing of the ablation. Patients with red triangles are those where recurrence was diagnosed after the final measurement. (**B**) Kaplan-Meier curves of recurrence by pre-ablation PIVKA-II and AFP levels. Pre-ablation PIVKA-II levels were dichotomised based on the median value. Pre-ablation AFP levels were dichotomised based on AFP secretor/non-secretor status. *P*-values are from log-rank tests.

All patients achieved a complete response to their radiofrequency ablation on their post-ablation imaging by mRECIST criteria. In 10/13 patients (77%), the serial PIVKA-II levels showed a concordant response, with a reduction in the PIVKA-II levels post ablation in either the first or subsequent samples (Figure 3A). Serum AFP levels were less informative as 6 patients were AFP non-secretors. Of the remainder, 5/7 (71%) patients demonstrated a concordant response, with declining AFP levels following ablation.

Four patients developed a recurrence during follow-up at 3, 7, 10 and 17 months post-ablation, respectively. In all four patients the serum PIVKA-II levels showed a concordant response with a rise in PIVKA-II levels (following an initial reduction in three patients corresponding with initial response) (Figure 3A). In all four patients, the rise in PIVKA-II levels predated radiological progression. The AFP levels increased progressively from baseline in two of the patients who subsequently progressed, decreased and then increased prior to progression in one patient and the final patient was an AFP non-secretor.

Patients who developed recurrence tended to have higher baseline PIVKA-II levels, compared to those who did not recur. To assess this further, patients were divided into two groups based on their baseline PIVKA-II levels, using the median as a cut-off value. Of those with baseline PIVKA-II levels of less than 125 mAU/mL (*n* = 6), no patients developed recurrence during the follow up period. However, for those with PIVKA-II of 125 mAU/mL or more (*n* = 7), a total of four developed recurrence, giving a Kaplan-Meier estimated recurrence rate of 100% after 17 months of follow up (Figure 3B). Despite this, no significant difference was detected between the groups (*p* = 0.097), likely as a result of low statistical power due to the small sample size. A weaker association between baseline AFP levels and recurrence was detected (*p* = 0.395, Figure 3B), with Kaplan-Meier estimated rates at one year of 36% vs. 50% for AFP secretors (*n* = 7) vs. AFP non-secretors (*n* = 6).

### PIVKA-II and AFP levels in patients undergoing transarterial chemoembolisation

A total of 36 patients planned for initial TACE were enrolled (20 as bridging therapy for early stage HCC and 16 as palliative treatment for intermediate stage HCC). Altogether 58 TACE procedures were performed in this cohort; 18 patients underwent a single TACE procedure, 14 patients underwent two procedures and 4 patients underwent three TACE procedures. The baseline samples were taken a median of 30 days before the first TACE procedure and serial PIVKA-II and AFP levels were measured approximately every three months thereafter, to coincide with routine clinic visits. The median number of samples collected per patient was 3 (range 2–6).

In six patients, the time frame between the baseline and the first follow up measurement was greater than four months, thus these patients were excluded from further analysis. An additional patient rapidly progressed clinically and one patient underwent subsequent liver transplantation before any post-TACE imaging, thus these two patients were also excluded from further analysis. Of the remaining 28 patients, following their first TACE procedure, 7 patients (25%) achieved a complete response by mRECIST criteria, 18 patients (64%) achieved a partial response by mRECIST criteria and 3 patients (11%) demonstrated no response by mRECIST criteria.

### Complete response

**Figure 4:**
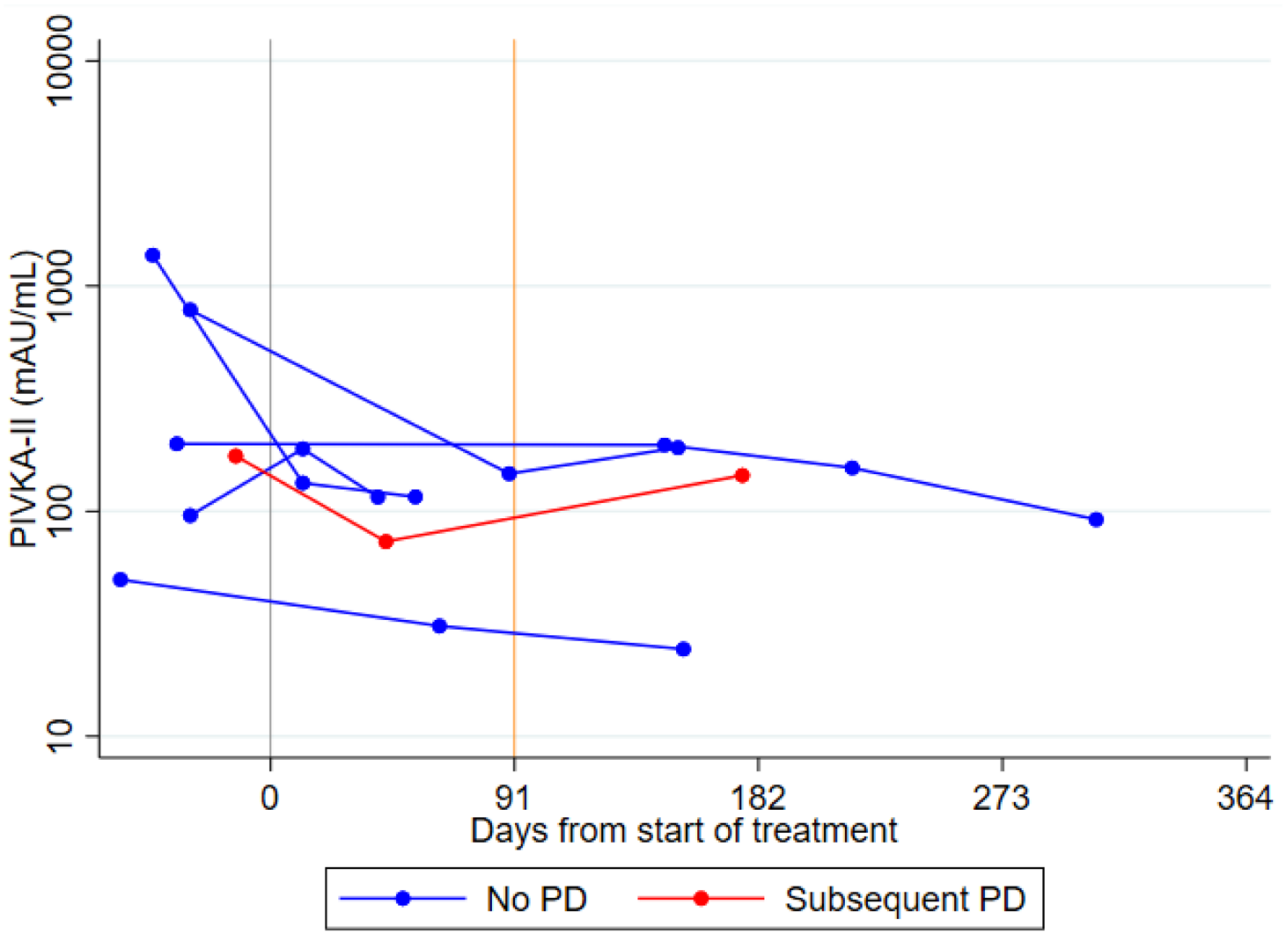
Spider plots of longitudinal PIVKA-II levels in TACE patients achieving a complete response. A log_10_ scale has been used for the PIVKA-II values.

Of the 7 patients who achieved a radiological complete response, the serial PIVKA-II levels showed a concordant response in 6 patients, with decreasing levels on the first or subsequent samples post-TACE (Figure 4). The remaining patient had a very low PIVKA-II level (32.5 mAU/mL), which remained unchanged – likely representing a PIVKA-II non-secretor. One patient developed progressive disease nine months after their initial TACE and their serum PIVKA-II levels showed a corresponding increase (following an initial reduction) that predated radiological progression.

Four patients were AFP non-secretors, but in the remaining three patients, the serial AFP levels also demonstrated a concordant response, with a continuous decline post-TACE (one of these patients included the potential PIVKA-II non-secretor).

### Partial response

Of the 18 patients who achieved a partial response, 11 patients (61%) showed a concordant PIVKA-II response, with continuously decreasing PIVKA-II levels following initial TACE. Three of these partial responders subsequently demonstrated evidence of progressive disease and in all three patients the serum PIVKA-II level demonstrated a corresponding increase, which again predated radiological progression. The AFP response was less concordant among these 11 patients: four patients were AFP non-secretors and only four (50%) of the remaining patients demonstrated a concordant AFP response, with decreasing levels post TACE.

Three of the 18 patients (17%) who achieved a partial response demonstrated no change in serial PIVKA-II levels. All three of these patients were also AFP non-secretors.

In the remaining four patients who achieved a partial response (22%), a discordant response was observed with rising serum PIVKA-II levels despite radiological improvement. Interestingly, one of these four discordant patients underwent a second TACE and achieved a near complete response by mRECIST criteria and their serum PIVKA-II level decreased correspondingly after their second TACE. The remaining three patients who demonstrated a rising PIVKA-II level also underwent a second TACE procedure but progressed or demonstrated extensive residual active disease; their serum PIVKA-II levels continued to rise post-second TACE, indicating that rising PIVKA-II levels post TACE may predict poor outcomes. Serum AFP levels were again less informative with three of the four PIVKA-II discordant patients being AFP non-secretors and the remaining patient demonstrating discordant falling levels, despite subsequent radiological progression.

### PIVKA-II and AFP levels in patients undergoing treatment with Sorafenib

A total of 35 patients who commenced Sorafenib for advanced HCC were enrolled. The baseline sample was taken a median of 1 day before the commencement of Sorafenib and serial PIVKA-II and AFP levels were measured approximately every month thereafter, to coincide with routine clinic visits. The median number of samples collected per patient was 3 (range 2–10).

**Figure 5:**
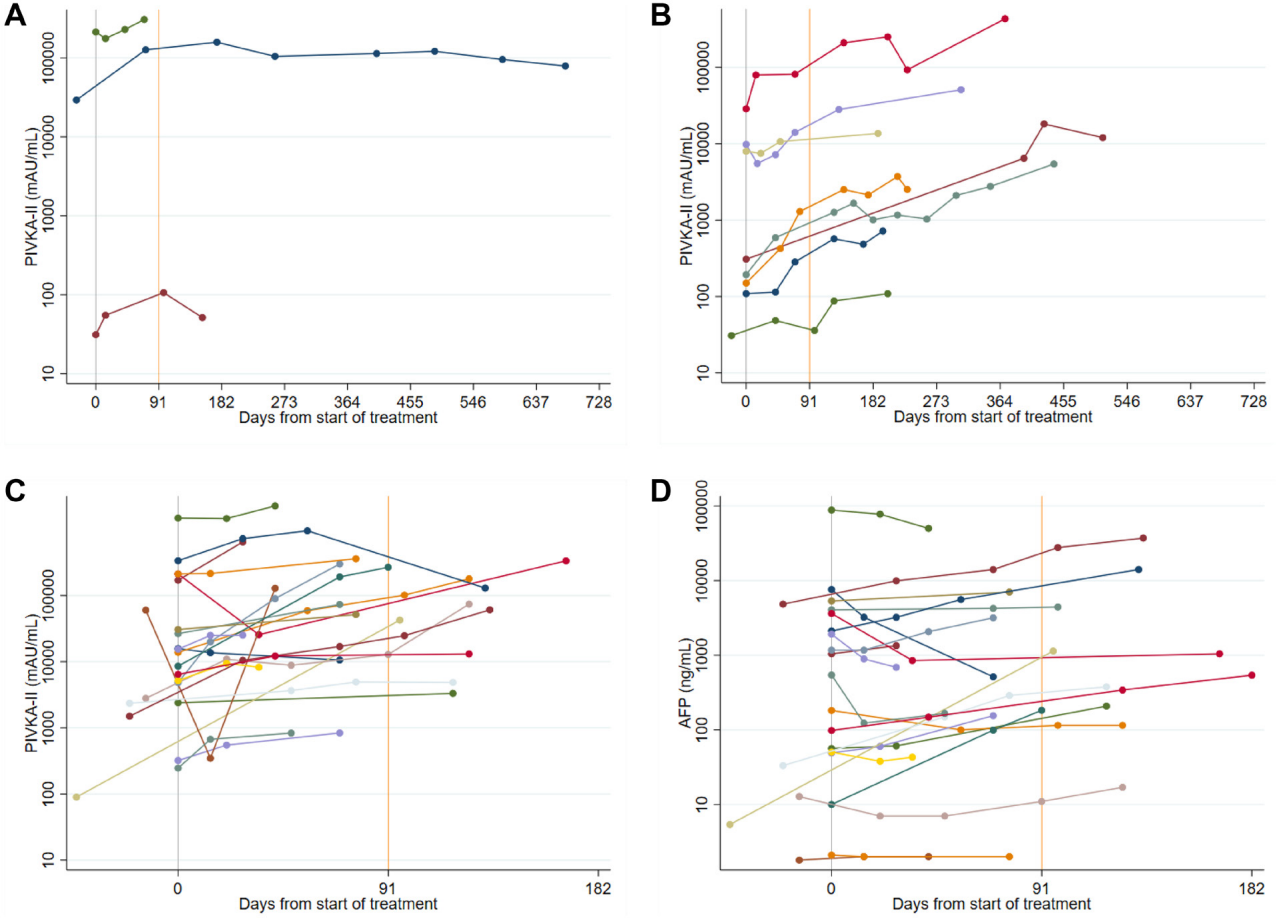
(**A**) Spider plots of longitudinal PIVKA-II levels in Sorafenib patients: stable disease. A log_10_ scale has been used for the PIVKA-II values. (**B**) Spider plots of longitudinal PIVKA-II levels in Sorafenib patients: stable disease followed by progression. A log_10_ scale has been used for the PIVKA-II values. (**C** and **D**) Spider plots of longitudinal PIVKA-II and AFP levels in Sorafenib patients: progressive disease. A log_10_ scale has been used for the PIVKA-II and AFP values.

Five patients achieved stable disease with no subsequent change. Of these, one patient was subsequently lost to follow up and one patient developed liver decompensation and discontinued treatment. The remaining three patients maintained disease stability until the end of follow up and their serial PIVKA-II levels showed a concordant response with stable readings throughout (Figure 5A). Serum AFP levels were similarly concordant in two patients (the remaining patient was an AFP non secretor).

An additional eight patients achieved stable disease as their best response but progressed at a later date. In all eight patients, PIVKA-II levels increased at the time of progression – five patients demonstrated a continuous rise in serum PIVKA-II levels, whereas in three patients the levels were initially stable, concordant with radiological response, but increased just prior to radiological progression (Figure 5B). Serum AFP levels were less informative, with three AFP non-secretors. The remaining five patients showed similar concordant changes, with continuously rising AFP levels in three patients and stable followed by rising AFP levels in two patients.

The remaining 22 patients achieved progressive disease as their best response. Serial PIVKA-II levels showed a concordant response in all but one patient, with continuously rising levels during follow up (Figure 5C). Conversely, serum AFP levels showed a concordant rise in only 13/22 patients (60%). In a further three patients, serum AFP levels actually decreased, in another three patients serum AFP levels remained stable despite radiological progression, and the remaining three patients were AFP non-secretors (Figure 5D).

### Combining PIVKA-II and AFP levels

A concordant response was recorded if concordant changes were observed with both PIVKA-II and AFP levels (or if one was a non-secretor). Combining both biomarkers did not improve the predictive accuracy of PIVKA-II alone (Table 3).

**Table 3:** Summary of the tumour marker trends compared to radiological response

Treatment	PIVKA-II		AFP			PIVKA-II & AFP	
	Concordant change (%)	Discordant change (%)	Non-secretors (%)	Concordant change (%)	Discordant change (%)	Concordant change (%)	Discordant change (%)
Ablation (*n* = 13)	10 (77%)	3 (23%)	6 (46%)	5/7 (71%)	2/7 (29%)	10 (77%)	3 (23%)
TACE (*n* = 28)	18 (64%)	10 (36%)^*^	16 (57%)	8/12 (67%)	4/12 (33%)	19 (68%)	9 (32%)
Sorafenib (*n* = 35)	27 (77%)	8 (23%)	8 (23%)	15/27 (56%)	12/27 (44%)	24 (69%)	11 (31%)

Analysis of trends in AFP excludes non-secretors; hence rates are based on the stated denominators. ^*^includes 5 patients (18%) who demonstrated no change rather than a discordant change.

### AFP non-secretors

Across the three treatment groups, a total of 30/76 patients (39%) were AFP non-secretors (Table 3). Of these, serum PIVKA-II levels demonstrated a concordant response following treatment in 18 patients (60%).

## DISCUSSION

To our knowledge, this is the first prospective study evaluating PIVKA-II levels following HCC treatment in a UK population. Our results show that changes in serum PIVKA-II levels track concordantly with treatment response in the majority of patients, across a wide range of treatment modalities. Our results in a Western cohort are consistent with the previous findings reported from Asian cohorts and are also consistent with a recent prospective study from a European cohort [14–18, 26, 27]. Collectively, the available evidence therefore confirms the value of PIVKA-II as a biomarker of tumour response across all stages of HCC, irrespective of disease aetiology. This has also been demonstrated for PIVKA-II (as part of the GALAD model) in the diagnostic setting [12].

Whilst AFP has been commonly used as a serum biomarker of tumour response for HCC for many years, one of its major limitations is that approximately 30–50% of patients with HCC are AFP non-secretors [28, 29]. We found that 40% of our prospective cohort were AFP non-secretors and, among this group, we observed that PIVKA-II was a useful alternative serum biomarker, with changes in levels tracking concordantly with treatment response in 60% of AFP non-secretors.

Our results also show the improved performance of PIVKA-II compared to AFP as a biomarker of tumour response. This finding has been observed by other authors and is not surprising as PIVKA-II is intimately linked to cancer development and growth. PIVKA-II binds to the hepatocyte growth factor receptor, c-MET, leading to tumour growth, invasion and tumour metastases via the JAK-1/STAT3 and ERK 1/2 MAPK signalling pathways, [30, 31] whereas AFP is thought to be more a marker for hepatic progenitor cells or their subtypes [32]. This is also the likely explanation for the rarity of patients who may be considered PIVKA-II non-secretors.

Nearly all the prospective studies to date have assessed changes in PIVKA-II levels only immediately post treatment. A major strength of our study is the longer duration of follow up (often until disease progression) and, consequently, we were able to observe that rises in PIVKA-II levels almost always predated radiological progression. This has previously been reported for PIVKA-II in the diagnostic setting, where levels of PIVKA-II have been found to be elevated over one year before HCC was detected radiologically, but has not previously been reported in patients on treatment [33]. If this finding is confirmed in subsequent larger prospective studies, this has the potential to be of great clinical significance and could be used to help switch patients to subsequent treatment earlier, before they deteriorate clinically. Population-based studies have observed that currently, only a small proportion of patients are able to receive second-line therapies [34].

Nevertheless, whilst demonstrating an improvement over AFP, the performance of PIVKA-II as a single serum biomarker is still limited. This was also observed in the diagnostic setting and the best performance (with the highest sensitivity and specificity) has been observed only with the combination of three biomarkers (AFP, AFP-L3 and PIVKA-II) as part of the GALAD score [12]. One of the main limitations of our study is the small sample size and larger prospective studies are thus needed but we would recommend that future studies explore the GALAD score as a biomarker of tumour response, rather than PIVKA-II in isolation.

PIVKA-II has also been shown to be strongly associated with tumour biology, with higher levels significantly related to the presence of vascular invasion, larger tumour size and poorer differentiation [33, 35]. Our results also add to the evidence base, with higher baseline PIVKA-II levels associated with increasing tumour stage, larger tumour size and poorer differentiation.

Microvascular invasion is known to be a major predictor of tumour recurrence after curative liver resection or orthotopic liver transplantation [20, 21]. *In vitro*, PIVKA-II has been shown to induce over-expression of epithelial growth factor receptor and vascular endothelial growth factor, directly stimulating the proliferation and migration of vascular endothelial cells [36–38]. We observed that higher pre-transplant levels of serum PIVKA-II predicted for microvascular invasion, which is consistent with the results from previous studies from Asia and a recent cohort from France [19–21, 39]. Collectively, the data thus confirms the value of pre-surgical levels of PIVKA-II in predicting microvascular invasion, again irrespective of disease aetiology. Future prospective studies are now needed to assess the role of pre-operative levels of PIVKA-II in selecting patients for surgical treatment and/or selecting patients for adjuvant therapy, in addition to its utility in switching between therapies.

## MATERIALS AND METHODS

### Study population

Patients with early, intermediate or advanced stage HCC (according to the Barcelona-clinic Liver Cancer Classification (BCLC)) who were due to commence standard treatment (liver transplantation, radiofrequency ablation, transarterial chemoembolisation or Sorafenib) were prospectively recruited between March 2016 and March 2018 at the Queen Elizabeth Hospital Birmingham, United Kingdom. All patients were 18 years of age or older with a confirmed diagnosis of HCC, either histologically or radiologically, as per the internationally accepted AASLD criteria. Patients were excluded if they were on the anticoagulant warfarin, had co-existing malignancy, or were pregnant. The study was approved by the National Research Ethics Committee (REC reference 06/Q2707/182) and all patients provided written informed consent before inclusion and any study-related procedures.

### Data collection

Data was collected on baseline demographics, aetiology of underlying liver disease, Child-Pugh score, laboratory test results and tumour characteristics. In transplanted patients, histological findings from the liver explant (including tumour differentiation and the presence of microvascular invasion, defined as microscopic invasion of small vessels by tumour) were also collected.

### Serum sample collection

Peripheral blood samples for measurement of serum PIVKA-II and AFP levels were taken before treatment (baseline) and at the time points detailed below following treatment, until progression or the end of follow up (December 2018). The time points were chosen to coincide with routine clinical review:

*Liver transplantation*: at a subsequent follow up appointment following transplantation (before any recurrence).

*Radiofrequency ablation:* at approximately three monthly intervals following ablation.

*Transarterial chemoembolisation*: four to six weeks post TACE and then at approximately three monthly intervals.

*Sorafenib*: at approximately monthly intervals following commencement of Sorafenib.

### Processing of serum samples

10 ml of whole blood were collected from patients using a serum separator tube and allowed to clot for one hour at room temperature. Samples were then centrifuged for 20 minutes at 3000 g and the serum extracted. Aliquots of serum were immediately frozen and stored at −80°C until analysis.

### Measurement of serum PIVKA-II and AFP levels

Serum levels of PIVKA-II and AFP were measured using the Tosoh Automated Enzyme Immunoassay Analyzer AIA-900 kindly provided by Tosoh Bioscience, following the manufacturer’s instructions. The Tosoh AIA-900 analyser uses a two-step immunoenzymetric assay. PIVKA-II non-secretors were defined as patients with serum PIVKA-II levels persistently below 40 mAU/mL. AFP non-secretors were defined as patients with serum AFP levels persistently below 20 ng/mL.

### Statistical analysis

Comparisons of AFP and PIVKA-II across cohort characteristics were performed using Spearman’s (rho) correlation coefficients for continuous variables, and Mann-Whitney *U* tests for dichotomous variables. Nominal variables with more than two categories were analysed using Kruskal-Wallis tests, whilst ordinal variables were analysed using Jonckheere-Terpstra tests. For the subgroup of patients undergoing ablation therapy, recurrence rates were estimated using Kaplan-Meier curves, in order to account for the follow-up time, with comparisons between groups performed using log-rank tests. In patients with serial AFP and PIVKA-II measurements in the post-treatment period, trajectories were visualised with spider plots.

All analyses were performed using IBM SPSS 22 (IBM Corp. Armonk, NY, USA), with *p* < 0.05 deemed to be indicative of statistical significance throughout.

## SUPPLEMENTARY MATERIALS



## References

[1] Sung H , Ferlay J , Siegel RL , Laversanne M , Soerjomataram I , Jemal A , Bray F . Global Cancer Statistics 2020: GLOBOCAN Estimates of Incidence and Mortality Worldwide for 36 Cancers in 185 Countries. CA Cancer J Clin. 2021; 71:209–49. 10.3322/caac.21660. 33538338

[2] Singal AG , Lampertico P , Nahon P . Reply to: "Challenges associated with the roll-out of HCC surveillance in sub-Saharan Africa - the case of Uganda". J Hepatol. 2020; 73:1273–74. 10.1016/j.jhep.2020.07.003. 32800586

[3] Cross TJS , Villanueva A , Shetty S , Wilkes E , Collins P , Adair A , Jones RL , Foxton MR , Meyer T , Stern N , Warshow U , Khan N , Prince M , et al. A national survey of the provision of ultrasound surveillance for the detection of hepatocellular carcinoma. Frontline Gastroenterol. 2016; 7:82–89. 10.1136/flgastro-2015-100617. 28840911 PMC5369506

[4] Heimbach JK , Kulik LM , Finn RS , Sirlin CB , Abecassis MM , Roberts LR , Zhu AX , Murad MH , Marrero JA . AASLD guidelines for the treatment of hepatocellular carcinoma. Hepatology. 2018; 67:358–80. 10.1002/hep.29086. 28130846

[5] European Association for the Study of the Liver. EASL Clinical Practice Guidelines: Management of hepatocellular carcinoma. J Hepatol. 2018; 69:182–36. 10.1016/j.jhep.2018.03.019. 29628281

[6] Bruix J , Sherman M , and American Association for the Study of Liver Diseases. Management of hepatocellular carcinoma: an update. Hepatology. 2011; 53:1020–22. 10.1002/hep.24199. 21374666 PMC3084991

[7] Liebman HA , Furie BC , Tong MJ , Blanchard RA , Lo KJ , Lee SD , Coleman MS , Furie B . Des-gamma-carboxy (abnormal) prothrombin as a serum marker of primary hepatocellular carcinoma. N Engl J Med. 1984; 310:1427–31. 10.1056/NEJM198405313102204. 6201741

[8] Li C , Zhang Z , Zhang P , Liu J . Diagnostic accuracy of des-gamma-carboxy prothrombin versus α-fetoprotein for hepatocellular carcinoma: A systematic review. Hepatol Res. 2014; 44:E11–25. 10.1111/hepr.12201. 23834468

[9] Xing H , Zheng YJ , Han J , Zhang H , Li ZL , Lau WY , Shen F , Yang T . Protein induced by vitamin K absence or antagonist-II versus alpha-fetoprotein in the diagnosis of hepatocellular carcinoma: A systematic review with meta-analysis. Hepatobiliary Pancreat Dis Int. 2018; 17:487–95. 10.1016/j.hbpd.2018.09.009. 30257796

[10] Toyoda H , Kumada T , Osaki Y , Oka H , Urano F , Kudo M , Matsunaga T . Staging hepatocellular carcinoma by a novel scoring system (BALAD score) based on serum markers. Clin Gastroenterol Hepatol. 2006; 4:1528–36. 10.1016/j.cgh.2006.09.021. 17162244

[11] Johnson PJ , Pirrie SJ , Cox TF , Berhane S , Teng M , Palmer D , Morse J , Hull D , Patman G , Kagebayashi C , Hussain S , Graham J , Reeves H , Satomura S . The detection of hepatocellular carcinoma using a prospectively developed and validated model based on serological biomarkers. Cancer Epidemiol Biomarkers Prev. 2014; 23:144–53. 10.1158/1055-9965.EPI-13-0870. 24220911

[12] Berhane S , Toyoda H , Tada T , Kumada T , Kagebayashi C , Satomura S , Schweitzer N , Vogel A , Manns MP , Benckert J , Berg T , Ebker M , Best J , et al. Role of the GALAD and BALAD-2 Serologic Models in Diagnosis of Hepatocellular Carcinoma and Prediction of Survival in Patients. Clin Gastroenterol Hepatol. 2016; 14:875–86.e6. 10.1016/j.cgh.2015.12.042. 26775025

[13] Kokudo N , Takemura N , Hasegawa K , Takayama T , Kubo S , Shimada M , Nagano H , Hatano E , Izumi N , Kaneko S , Kudo M , Iijima H , Genda T , et al. Clinical practice guidelines for hepatocellular carcinoma: The Japan Society of Hepatology 2017 (4th JSH-HCC guidelines) 2019 update. Hepatol Res. 2019; 49:1109–13. 10.1111/hepr.13411. 31336394

[14] Park WH , Shim JH , Han SB , Won HJ , Shin YM , Kim KM , Lim YS , Lee HC . Clinical utility of des-γ-carboxyprothrombin kinetics as a complement to radiologic response in patients with hepatocellular carcinoma undergoing transarterial chemoembolization. J Vasc Interv Radiol. 2012; 23:927–36. 10.1016/j.jvir.2012.04.021. 22633621

[15] Lee MH , Kim SU , Kim DY , Ahn SH , Choi EH , Lee KH , Lee DY , Seong J , Han KH , Chon CY , Park JY . Early on-treatment predictions of clinical outcomes using alpha-fetoprotein and des-gamma-carboxy prothrombin responses in patients with advanced hepatocellular carcinoma. J Gastroenterol Hepatol. 2012; 27:313–22. 10.1111/j.1440-1746.2011.06867.x. 21793906

[16] Lee YK , Kim SU , Kim DY , Ahn SH , Lee KH , Lee DY , Han KH , Chon CY , Park JY . Prognostic value of α-fetoprotein and des-γ-carboxy prothrombin responses in patients with hepatocellular carcinoma treated with transarterial chemoembolization. BMC Cancer. 2013; 13:5. 10.1186/1471-2407-13-5. 23282286 PMC3545962

[17] Arai T , Kobayashi A , Ohya A , Takahashi M , Yokoyama T , Shimizu A , Motoyama H , Furusawa N , Notake T , Kitagawa N , Sakai H , Imamura H , Kadoya M , Miyagawa S . Assessment of treatment outcomes based on tumor marker trends in patients with recurrent hepatocellular carcinoma undergoing trans-catheter arterial chemo-embolization. Int J Clin Oncol. 2014; 19:871–79. 10.1007/s10147-013-0634-6. 24218280

[18] Ueshima K , Kudo M , Takita M , Nagai T , Tatsumi C , Ueda T , Kitai S , Ishikawa E , Yada N , Inoue T , Hagiwara S , Minami Y , Chung H , Sakurai T . Des-γ-carboxyprothrombin may be a promising biomarker to determine the therapeutic efficacy of sorafenib for hepatocellular carcinoma. Dig Dis. 2011; 29:321–25. 10.1159/000327570. 21829024

[19] Shirabe K , Itoh S , Yoshizumi T , Soejima Y , Taketomi A , Aishima S , Maehara Y . The predictors of microvascular invasion in candidates for liver transplantation with hepatocellular carcinoma-with special reference to the serum levels of des-gamma-carboxy prothrombin. J Surg Oncol. 2007; 95:235–40. 10.1002/jso.20655. 17323337

[20] Eguchi S , Takatsuki M , Hidaka M , Soyama A , Tomonaga T , Muraoka I , Kanematsu T . Predictor for histological microvascular invasion of hepatocellular carcinoma: a lesson from 229 consecutive cases of curative liver resection. World J Surg. 2010; 34:1034–38. 10.1007/s00268-010-0424-5. 20127241

[21] Yamashita Y , Tsuijita E , Takeishi K , Fujiwara M , Kira S , Mori M , Aishima S , Taketomi A , Shirabe K , Ishida T , Maehara Y . Predictors for microinvasion of small hepatocellular carcinoma ≤ 2 cm. Ann Surg Oncol. 2012; 19:2027–34. 10.1245/s10434-011-2195-0. 22203184

[22] Seo SI , Kim HS , Kim WJ , Shin WG , Kim DJ , Kim KH , Jang MK , Lee JH , Kim JS , Kim HY , Kim DJ , Lee MS , Park CK . Diagnostic value of PIVKA-II and alpha-fetoprotein in hepatitis B virus-associated hepatocellular carcinoma. World J Gastroenterol. 2015; 21:3928–35. 10.3748/wjg.v21.i13.3928. 25852278 PMC4385540

[23] Qin X , Tang G , Gao R , Guo Z , Liu Z , Yu S , Chen M , Tao Z , Li S , Liu M , Wang L , Hou L , Xia L , et al. A multicenter study on PIVKA reference interval of healthy population and establishment of PIVKA cutoff value for hepatocellular carcinoma diagnosis in China. Int J Lab Hematol. 2017; 39:392–401. 10.1111/ijlh.12639. 28318145

[24] Yan C , Hu J , Yang J , Chen Z , Li H , Wei L , Zhang W , Xing H , Sang G , Wang X , Han R , Liu P , Li Z , et al. Serum ARCHITECT PIVKA-II reference interval in healthy Chinese adults: Sub-analysis from a prospective multicenter study. Clin Biochem. 2018; 54:32–36. 10.1016/j.clinbiochem.2018.02.007. 29448045

[25] Bayart JL , Mairesse A , Gruson D , van Dievoet MA . Analytical performances and biological variation of PIVKA-II (des-y-carboxy-prothrombin) in European healthy adults. Clin Chim Acta. 2020; 509:264–67. 10.1016/j.cca.2020.06.035. 32589882

[26] Yang M , Zhang X , Liu J . Prognostic value of des-γ-carboxy prothrombin in patients with hepatocellular carcinoma treated with transarterial chemotherapy: A systematic review and meta-analysis. PLoS One. 2019; 14:e0225170. 10.1371/journal.pone.0225170. 31730646 PMC6857949

[27] Cerban R , Ester C , Iacob S , Paslaru L , Dumitru R , Grasu M , Constantin G , Popescu I , Gheorghe L . Evaluation of Tumor Response Using Alpha-fetoprotein and Des-gamma-carboxy Prothrombin in Hepatocellular Carcinoma Patients Who Underwent Transarterial Chemoembolization. Chirurgia (Bucur). 2018; 113:524–33. 10.21614/chirurgia.113.4.524. 30183583

[28] Carr BI , Buch SC , Kondragunta V , Pancoska P , Branch RA . Tumor and liver determinants of prognosis in unresectable hepatocellular carcinoma: a case cohort study. J Gastroenterol Hepatol. 2008; 23:1259–66. 10.1111/j.1440-1746.2008.05487.x. 18699979

[29] Carr BI , Pancoska P , Branch RA . Low alpha-fetoprotein hepatocellular carcinoma. J Gastroenterol Hepatol. 2010; 25:1543–49. 10.1111/j.1440-1746.2010.06303.x. 20796153

[30] Suzuki M , Shiraha H , Fujikawa T , Takaoka N , Ueda N , Nakanishi Y , Koike K , Takaki A , Shiratori Y . Des-gamma-carboxy prothrombin is a potential autologous growth factor for hepatocellular carcinoma. J Biol Chem. 2005; 280:6409–15. 10.1074/jbc.M406714200. 15582995

[31] Yue P , Gao ZH , Xue X , Cui SX , Zhao CR , Yuan Y , Yin Z , Inagaki Y , Kokudo N , Tang W , Qu XJ . Des-γ-carboxyl prothrombin induces matrix metalloproteinase activity in hepatocellular carcinoma cells by involving the ERK1/2 MAPK signalling pathway. Eur J Cancer. 2011; 47:1115–24. 10.1016/j.ejca.2011.01.017. 21349701

[32] Mizejewski GJ . Biological role of alpha-fetoprotein in cancer: prospects for anticancer therapy. Expert Rev Anticancer Ther. 2002; 2:709–35. 10.1586/14737140.2.6.709. 12503217

[33] Yu R , Tan Z , Xiang X , Dan Y , Deng G . Effectiveness of PIVKA-II in the detection of hepatocellular carcinoma based on real-world clinical data. BMC Cancer. 2017; 17:608. 10.1186/s12885-017-3609-6. 28863782 PMC5580438

[34] Tsang ES , Davies JM , Loree JM , Lim HJ , Renouf DJ , Gill S . Eligibility for second-line therapy in patients with advanced hepatocellular carcinoma (aHCC): A BC Cancer population-based study. J Clin Oncol. 2020 (Suppl 4); 38:491. 10.1200/JCO.2020.38.4_suppl.491. 32868523

[35] Park MS , Lee KW , Kim H , Choi YR , Hong G , Yi NJ , Suh KS . Usefulness of PIVKA-II After Living-donor Liver Transplantation for Hepatocellular Carcinoma. Transplant Proc. 2017; 49:1109–13. 10.1016/j.transproceed.2017.03.017. 28583537

[36] Fujikawa T , Shiraha H , Ueda N , Takaoka N , Nakanishi Y , Matsuo N , Tanaka S , Nishina S , Suzuki M , Takaki A , Sakaguchi K , Shiratori Y . Des-gamma-carboxyl prothrombin-promoted vascular endothelial cell proliferation and migration. J Biol Chem. 2007; 282:8741–48. 10.1074/jbc.M609358200. 17255102

[37] Wang SB , Cheng YN , Cui SX , Zhong JL , Ward SG , Sun LR , Chen MH , Kokudo N , Tang W , Qu XJ . Des-gamma-carboxy prothrombin stimulates human vascular endothelial cell growth and migration. Clin Exp Metastasis. 2009; 26:469–77. 10.1007/s10585-009-9246-y. 19263229

[38] Gao FJ , Cui SX , Chen MH , Cheng YN , Sun LR , Ward SG , Kokudo N , Tang W , Qu XJ . Des-gamma-carboxy prothrombin increases the expression of angiogenic factors in human hepatocellular carcinoma cells. Life Sci. 2008; 83:815–20. 10.1016/j.lfs.2008.10.003. 18976674

[39] Poté N , Cauchy F , Albuquerque M , Voitot H , Belghiti J , Castera L , Puy H , Bedossa P , Paradis V . Performance of PIVKA-II for early hepatocellular carcinoma diagnosis and prediction of microvascular invasion. J Hepatol. 2015; 62:848–54. 10.1016/j.jhep.2014.11.005. 25450201

