# Supplementary material for: The potential of PIVKA-II as a treatment response biomarker in hepatocellular carcinoma: a prospective United Kingdom cohort study
Source: Oncotarget. 2021 Nov 23;12(24):2338–50. doi: 10.18632/oncotarget.28136 (PMC8629402; doi:10.18632/oncotarget.28136)
Supplement: Supplementary file 1 [file oncotarget-12-2338-s001.pdf]

The potential of PIVKA-II as a treatment response biomarker in hepatocellular carcinoma: a prospective United Kingdom cohort study

SUPPLEMENTARY MATERIALS

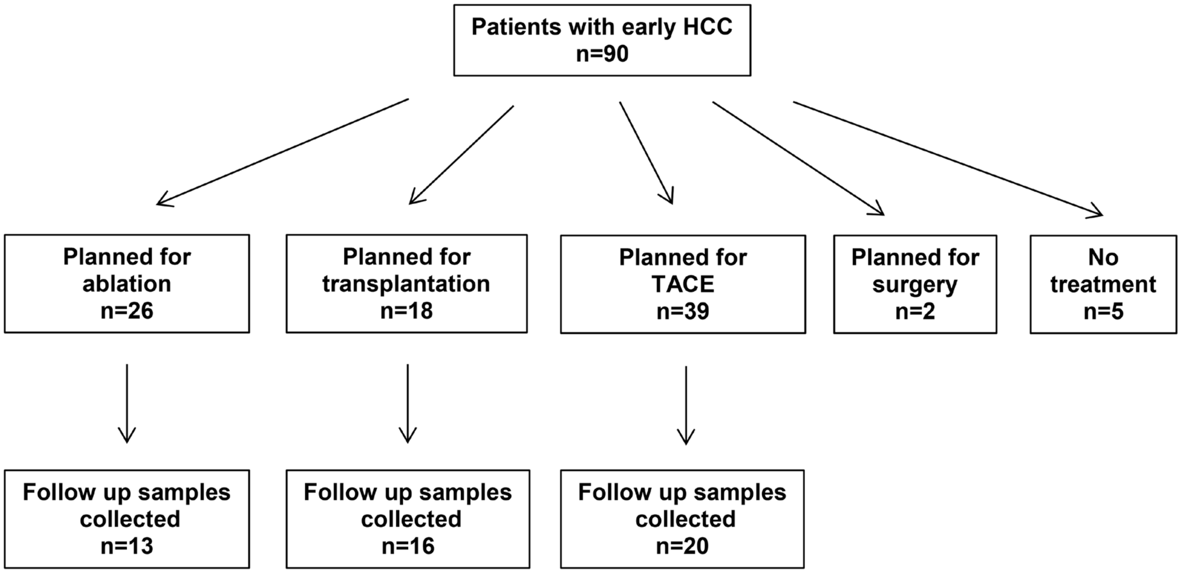

Supplementary Figure 1: Treatment flow for patients with early HCC.
